# Comprehensive *in vivo* Mapping of the Human Basal Ganglia and Thalamic Connectome in Individuals Using 7T MRI

**DOI:** 10.1371/journal.pone.0029153

**Published:** 2012-01-03

**Authors:** Christophe Lenglet, Aviva Abosch, Essa Yacoub, Federico De Martino, Guillermo Sapiro, Noam Harel

**Affiliations:** 1 Center for Magnetic Resonance Research, Department of Radiology, University of Minnesota, Minneapolis, Minnesota, United States of America; 2 Department of Neurosurgery, University of Minnesota, Minneapolis, Minnesota, United States of America; 3 Department of Cognitive Neuroscience, Maastricht University, Maastricht, The Netherlands; 4 Department of Electrical and Computer Engineering, University of Minnesota, Minneapolis, Minnesota, United States of America; University of California San Francisco, United States of America

## Abstract

Basal ganglia circuits are affected in neurological disorders such as Parkinson's disease (PD), essential tremor, dystonia and Tourette syndrome. Understanding the structural and functional connectivity of these circuits is critical for elucidating the mechanisms of the movement and neuropsychiatric disorders, and is vital for developing new therapeutic strategies such as deep brain stimulation (DBS). Knowledge about the connectivity of the human basal ganglia and thalamus has rapidly evolved over recent years through non-invasive imaging techniques, but has remained incomplete because of insufficient resolution and sensitivity of these techniques. Here, we present an imaging and computational protocol designed to generate a comprehensive *in vivo* and subject-specific, three-dimensional model of the structure and connections of the human basal ganglia. High-resolution structural and functional magnetic resonance images were acquired with a 7-Tesla magnet. Capitalizing on the enhanced signal-to-noise ratio (SNR) and enriched contrast obtained at high-field MRI, detailed structural and connectivity representations of the human basal ganglia and thalamus were achieved. This unique combination of multiple imaging modalities enabled the *in-vivo* visualization of the individual human basal ganglia and thalamic nuclei, the reconstruction of seven white-matter pathways and their connectivity probability that, to date, have only been reported in animal studies, histologically, or group-averaged MRI population studies. Also described are subject-specific parcellations of the basal ganglia and thalamus into sub-territories based on their distinct connectivity patterns. These anatomical connectivity findings are supported by functional connectivity data derived from resting-state functional MRI (R-fMRI). This work demonstrates new capabilities for studying basal ganglia circuitry, and opens new avenues of investigation into the movement and neuropsychiatric disorders, in individual human subjects.

## Introduction

*In vivo* structural and connectivity modeling of the human basal ganglia is critical for understanding the movement and neuropsychiatric disorders. Such modeling is also vital for designing novel, and refining existing, therapeutic interventions for these disorders. Targeting during DBS surgery, for example, heavily relies on atlases constructed a century ago and comprised of a limited number of human *post-mortem* specimens. Access to a comprehensive, high-resolution, three-dimensional model of the relevant anatomy—i.e., one based on a patient's own brain—might significantly improve surgical outcome, shorten the procedure by enhancing surgical planning, and help shed new light on factors that could currently be affecting therapeutic results. In this paper we present a new imaging and computational protocol to build a subject-specific model of the basal ganglia and thalamic connectome, exploiting the enhanced signal-to-noise ratio (SNR), contrast, and resolution attainable by using high-field 7T MRI.

From the standpoint of anatomical connectivity, the majority of cortical output reaches the striatum—composed of the caudate nucleus (CN) and putamen (Pu)—the subthalamic nucleus (STN) and thalamus (Tha). Striatal output to Tha is then believed to pass through the main basal ganglia output nuclei [1], which are the substantia nigra pars reticulata (SNr) and internal segment of the globus pallidus (GPi). Projections of the striatum are organized into a direct and an indirect pathway. The direct pathway consists of monosynaptic projections from the striatum to GPi and SNr. In the indirect pathway, the external segment of the globus pallidus (GPe) and STN receive input from the striatum, and project to the output structures of the basal ganglia. The substantia nigra pars compacta (SNc), which receives input from GPe and projects to STN, projects massively to the striatum via the nigrostriatal pathway. Information about the interaction between the direct and indirect pathways is not fully comprehensive in normal or disease states [2]–[7], and the current model awaits further validation.

The anatomy of these basal ganglia connections suggested, at least in part, that these structures operate as part of recurrent circuits with the cerebral cortex [5]. The initial model for cortico-basal-ganglia-thalamic-cortex connectivity proposed parallel pathways sub-serving sensorimotor, associative, and limbic information processing [3], [8]. However, increasing data collected over the past quarter-century, in animals [9]–[11] or patients [12], [13], support the presence of complicated interconnections in these cortico-basal ganglia loops [5], [11], [14]–[16], with patterns of convergence and divergence within individual circuits that might be crucial for their function [5]. Such findings obligate a better understanding of the complete topology of these pathways.

Efforts aimed at validating these models have largely relied either on animal data [6], [11], [17], [18], *post-mortem* histological studies [19], or human *population* imaging studies—i.e., studies based on data that have been averaged over multiple subjects—in order to characterize subcortico-cortical structural [20]–[27] and functional [28]–[31] connectivity. Despite the valuable contribution of the studies cited above, a comprehensive description of the basal ganglia and thalamic circuitry is lacking, and, translating existing results derived from non-human primates (NHP) to humans is an arduous task [32]. Thus, a comprehensive study of *in vivo* basal ganglia circuitry in human subjects is needed.

Recent progress in high-field MRI [33] has enabled a re-evaluation of the anatomy and connectivity of brain regions. Using a high-field 7T MRI scanner, we reconstructed and characterized basal ganglia circuits previously identified only by histology or MRI population studies. Building on our previous work [33], the present study provides new information regarding (i) subject-specific *in-vivo* visualization and segmentation of basal ganglia and thalamus, (ii) comprehensive reconstructions of white matter pathways connecting these structures, (iii) quantification of the probability of each pathway, and (iv) identification of subdivisions of the basal ganglia and thalamus based on their anatomical connectivity patterns. The unique imaging and analysis protocol used in this study provided the exquisite level of detail required to successfully reconstruct seven pathways—including the pallidothalamic, subthalamopallidal and nigropallidal projections—all in individual subjects. In addition, the structural connectivity data presented here was supported by correlation with functional connectivity data obtained via resting-state fMRI.

## Results

**Figure 1:**
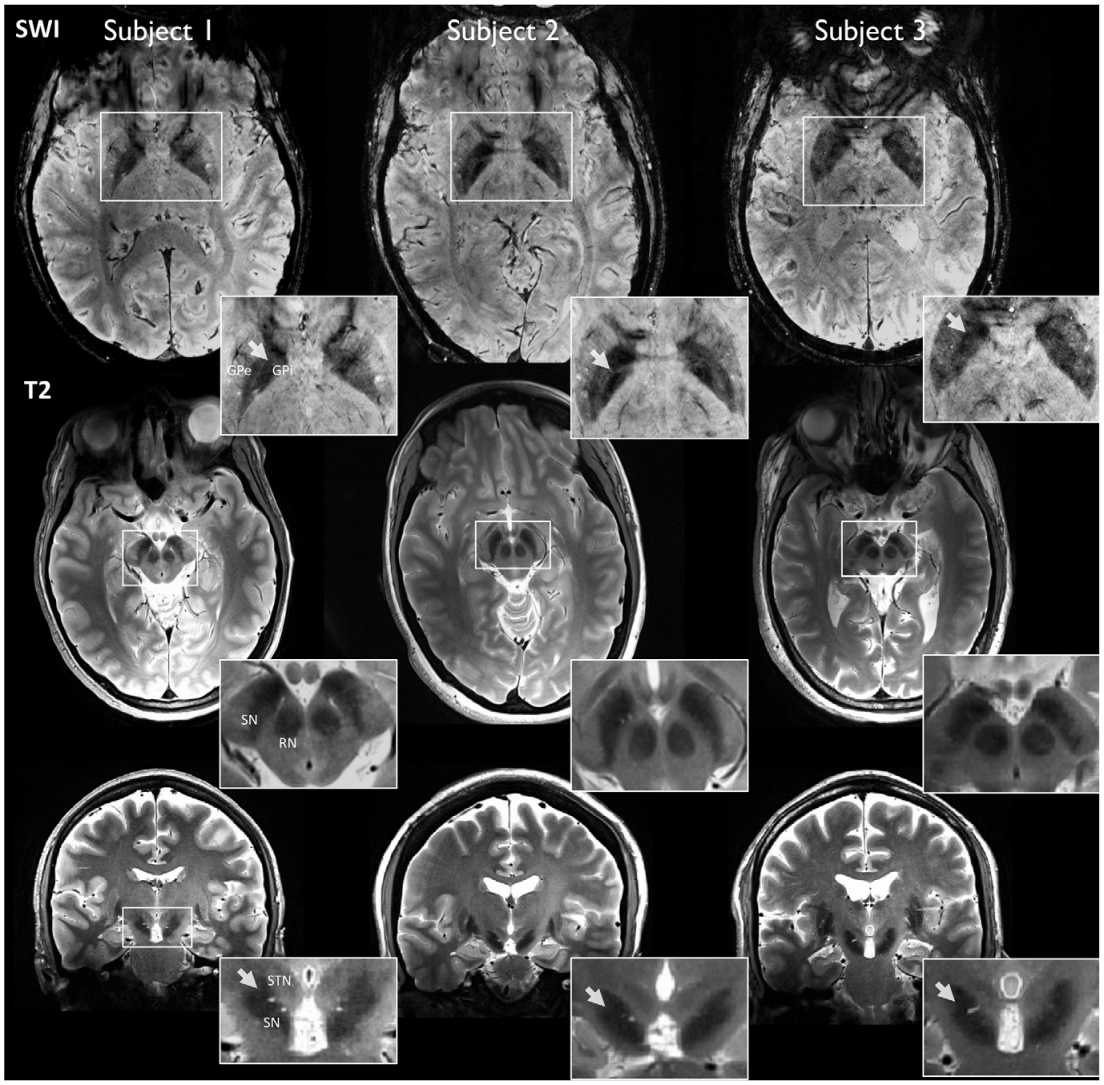
Structural imaging at 7T. (Top row) Axial high-resolution susceptibility-weighted images (SWI) in three subjects, at the level of the globus pallidus (GP), putamen and thalamus. 7T SWI provides high contrast between structures such as the external (GPe) and internal (GPi) segments of GP, as can be seen in each inset in the uppermost row. The white arrows indicate the border between GPe and GPi, known as the *lamina pallidi medialis* (uppermost insets). Axial high-resolution T2-weighted images in three subjects at the level of the substantia nigra (SN) and red nucleus (RN) are presented in the middle row. Coronal T2-weighted images in three subjects at the level of the subthalamic nucleus (STN) and SN (bottom row). Coronal images provide good contrast enabling differentiation between SN and STN along the lateral-medial axis, as indicated by the white arrow in each inset. SWI and T2-weighted images were co-registered and both used to segment GPe, GPi, SN, and STN. More details on the advantages of 7T SWI and T2-weighted imaging can be found in [33].

**Figure 2:**
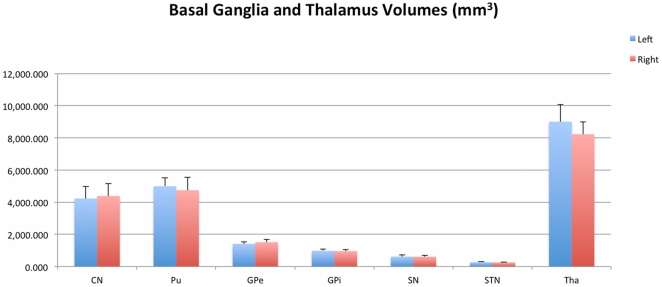
Volumes of segmented basal ganglia and thalamus. This graph summarizes statistics (mean and standard deviation over the five datasets) of the volume of the seven structures of interest in this study. Volumes were found to be in agreement with values reported in the literature. No statistical difference was detected between left and right hemispheres. Average agreement index (see Materials and Methods) was 0.94 for two datasets of the same subject acquired on different days. (Blue: left hemisphere, Red: right hemisphere).

**Figure 3:**
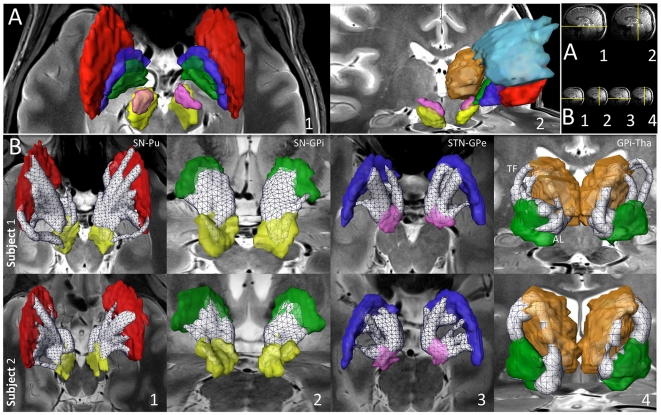
Segmentation and reconstruction of fiber pathways of the basal ganglia and thalamus. (Panel A) Three-dimensional visualization of manual segmentations of basal ganglia and thalamus from high-resolution SWI, T2-weighted images and fractional anisotropy maps. Segmentations are superimposed on T2-weighted images. (Panel B) White matter pathways identified using diffusion MRI probabilistic tractography. The white wireframe volumes represent white matter tracts identified as pathways of interest (see Materials and Methods). From left to right: (1) nigrostriatal, (2) nigropallidal, (3) subthalamopallidal and (4) pallidothalamic pathways with AL: *ansa lenticularis* and TF: *thalamic fasciculus*. Background images are 7T T2-weighted MR images. (Top right inset) Yellow lines depict the orientation and location of T2-weighted images in each panel. (A1) Axial image at the level of SN; (A2) Coronal image at the level of the posterior Tha; (B1) Axial image at the level of SN, caudo-rostral orientation; (B2) Coronal image at the level of the anterior GPi; (B3) Axial image slightly inferior to STN to avoid obscuring portions of the tract; (B4) Coronal image at the level of the posterior Tha, rostro-caudal orientation. Color code: Caudate nucleus, CN: light blue; Putamen, Pu: red; External globus pallidus, GPe: dark blue; Internal globus pallidus, GPi: green; Substantia nigra, SN: yellow; subthalamic nucleus, STN: magenta; Thalamus, Tha: orange.

Results were obtained from four healthy subjects, with a repeated data-acquisition session acquired in one of these subjects. A combination of high-resolution structural [33] (Fig. 1) and diffusion MRI was used to manually segment the seven structures of interest in each hemisphere (CN, Pu, GPe, GPi, SN, STN and Tha). The volumes of these structures are described in Fig. 2, and the corresponding three-dimensional visualization of each structure is presented in Fig. 3*A*. See Materials and Methods section for a detailed description of the steps required for data processing.

**Figure 4:**
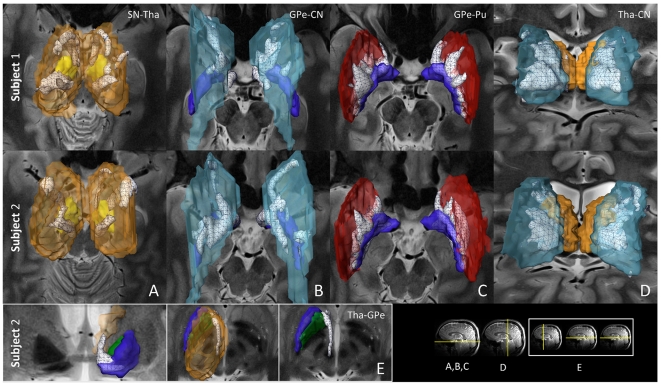
Reconstruction of fiber pathways of the basal ganglia and thalamus. White matter pathways identified using diffusion MRI probabilistic tractography. The white wireframe volumes represent white matter tracts identified as pathways of interest (see Materials and Methods). From left to right: (Panel A) nigrothalamic pathway, (Panel B) and (Panel C) pallidostriatal pathway, (Panel D) thalamostriatal pathway, (Panel E) thalamopallidal pathway. (Bottom right inset) Yellow lines depict the orientation and location of T2-weighted images in each panel. (Panels A, B, C) Axial images at the level of SN; (Panel D) Coronal image at the level of the posterior Tha; (Panel E, left) Coronal image at the level of the anterior thalamus; (Panel E, middle and right) Axial images at the level of the inferior GP. Color code: Caudate nucleus, CN: light blue; Putamen, Pu: red; External globus pallidus, GPe: dark blue; Internal globus pallidus, GPi: green; Substantia nigra, SN: yellow; Thalamus, Tha: orange.

**Figure 5:**
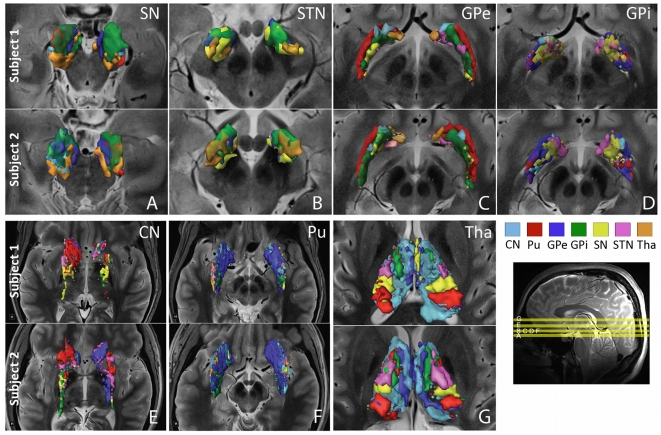
Parcellation of the basal ganglia and thalamus based on their white matter projections. Various sub-territories identified in the basal ganglia and thalamus by exploiting the fact that these divisions exhibit distinctively stronger connectivity with other structures. Within each of the seven regions-of-interest, voxels presenting a high probability of connection with another structure are categorized according to the color of that very structure. (Bottom right inset) The yellow lines depict the location of axial T2-weighted images in each panel. (Panel A) Inferior SN; (Panel B, C, D, F) Inferior GP; (Panel E) Superior GP; (Panel G) Superior Tha, inferior-superior view. Color code: Caudate nucleus, CN: light blue; Putamen, Pu: red; External globus pallidus, GPe: dark blue; Internal globus pallidus, GPi: green; Substantia nigra, SN: yellow; subthalamic nucleus, STN: magenta; Thalamus, Tha: orange.

**Figure 6:**
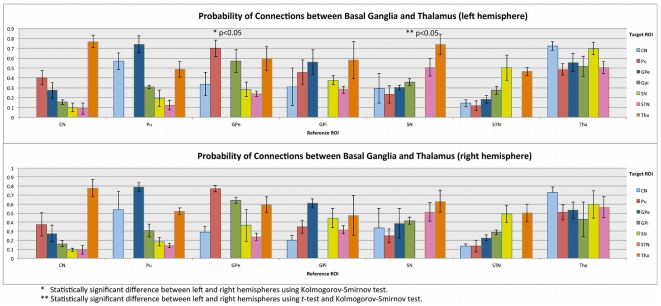
Probability of connection between basal ganglia and thalamus. This chart summarizes statistics (mean and standard deviation over the five datasets) of the proportion of probabilistic streamlines starting from each voxel of a given structure and reaching a specific target region, by comparison with the total number of streamlines reaching the entire basal ganglia area or thalamus. For a given subject, structure and target region, the proportion of probabilistic streamlines is defined as the average proportion over all voxels of the sub-territory of the structure connected to a specific target region. The sub-territory is defined as the area with proportion of streamline greater or equal to 50% of the maximum proportion. Color code: Caudate nucleus, CN: light blue; Putamen, Pu: red; External globus pallidus, GPe: dark blue; Internal globus pallidus, GPi: green; Substantia nigra, SN: yellow; subthalamic nucleus, STN: magenta; Thalamus, Tha: orange.

**Figure 7:**
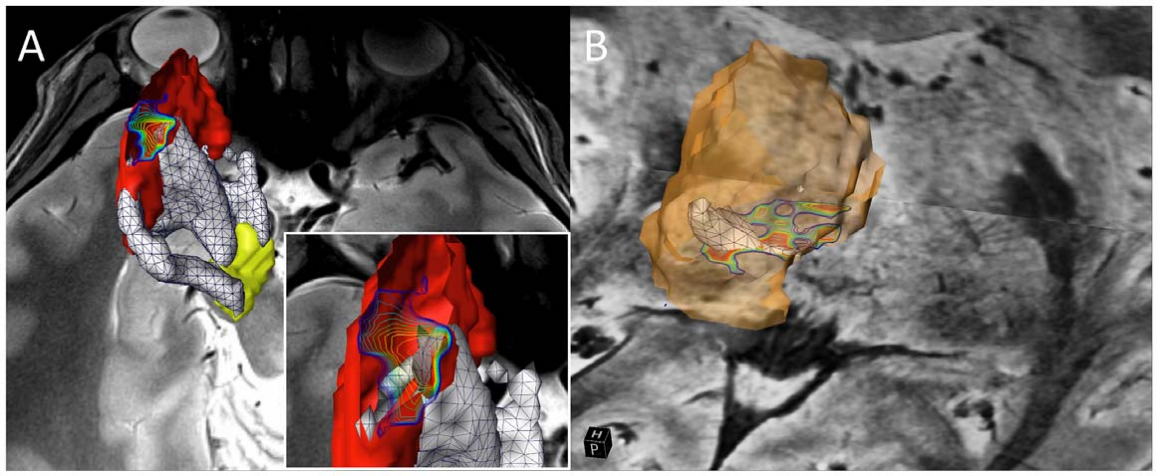
Spatial agreement between functional and anatomical connectivity maps. The figure demonstrates spatial overlap, within the putamen or thalamus, between the areas reached by white matter nigral projections and the resting-state functional activations of the substantia nigra. The white wireframe volumes represent reconstructions of the nigrostriatal (A) and nigrothalamic (B) pathways. R-fMRI activation maps of the substantia nigra were obtained using FDR (see Materials and Methods). They are represented using isolines of equal p-values, with blue lines corresponding to p = 0.01 and red lines to p≪0.01. Spatial agreement between anatomical and functional connectivity maps is visible in the dorsal part of the putamen (A) and in thalamus, within the putative Vc nucleus (B). Orientation in (A) is caudo-rostral with T2-weighted image at the level of the substantia nigra. Inset in (A) is a magnified view of the overlap area between anatomical and functional connectivity. The red surface representing putamen was clipped in its upper part to reveal the R-fMRI activation (isolines) and endpoint of the nigrostriatal projections. Orientation in (B) is oblique caudo-rostral with SWI images at the level of the inferior and anterior thalamus.

Three-dimensional visualization of basal ganglia and thalamic structures, their white matter pathways (Figs. 3*B* and 4), and the parcellation of the anatomical sub-territories obtained in individual subjects are presented in Fig. 5. The probability of each identified pathway is described in Fig. 6. Structural connectivity (diffusion) patterns are correlated with the functional connectivity of the seven structures of interest, as obtained by R-fMRI in Fig. 7.

We support our findings with well-established information derived from NHP and rodent studies and recent results from investigations of basal ganglia disorders in humans (Table 1).

**Table 1:** White matter pathways identified *in-vivo* in individual human subjects, and previous studies.

Pathways	Species	Year	Authors	Modalities
Nigrostriatal	Monkey	1972	Carpenter *et al.*	Lesions/staining
	Rat	1979	Beckstead *et al.*	Autoradiography
	Monkey	2008	Lehéricy *et al.*	MRI
Nigropallidal	Monkey	1972	Carpenter *et al.*	Lesions/staining
Nigrothalamic	Monkey	1972	Carpenter *et al.*	Lesions/staining
	Rat	1979	Beckstead *et al.*	Autoradiography
Subthalamopallidal	Monkey/Human	2000	Francois *et al.*	Tracing
	Monkey/Human	2004 *(review)*	Hamani *et al.*	Lesions/staining
	Human	2007	Aravamuthan *et al.*	MRI *(population)*
Pallidothalamic	Monkey/Rodent	1971 *(review)*	Kemp *et al.*	Light microscopy
	Monkey	1973	Kuo *et al.*	Lesions/staining
	Human (*post-mortem*)	2008	Gallay *et al.*	Staining/MRI
Striatopallidal	Monkey/Rodent	1971	Kemp *et al.*	Light microscopy
Thalamostriatal	Monkey	1995 *(review)*	Parent *et al.*	Lesions/staining

Previous studies demonstrated the existence of these seven white matter pathways using histology in animals or MRI population studies. Besides the comprehensive quantification of the probability of connection between all basal ganglia and thalamus, we successfully reconstructed and visualized these pathways in individual human subjects using high resolution 7T MRI.

### Volumes of the basal ganglia and thalamus, intra-observer variability

Estimated volumes of all segmented regions-of-interest (ROI; Fig. 2), across the five datasets, were found to be in agreement with values derived from MRI and histological reports [25], [34]–[41]. No statistical difference was detected between left and right hemispheres. Moreover, datasets acquired two days apart for Subject 3 were used to evaluate the reproducibility of segmentations (i.e. the performance of the segmentation expert, or “*intra-observer variability*”). The mean agreement index (AI, defined as: AI = 1−(|V_1_−V_2_|/(0.5*(V_1_+V_2_))), where V_1_ and V_2_ are the measured volumes on Day 1 and Day 2) was 0.94, across the seven structures and both hemispheres. More specifically, AI was slightly lower in the right hemisphere (0.93) than the left hemisphere (0.95), which could be due to lower contrast in the right hemisphere because of coil sensitivity variations, and/or poorer performance of the segmentation operator on this side. Our intra-observer AI is in agreement with, or outperforms, previous results on manual and automatic segmentation methods of brain structures, with typical AI values of 0.9 or less [42], [43].

### White matter tractography of the human basal ganglia and thalamus

Using probabilistic tractography (see details in Materials and Methods), we reconstructed the pathways connecting each pair of structures-of-interest—21, in total, as we reconstructed undirected pathways for each individual region-of-interest with the six remaining regions. Highly reproducible patterns of connectivity were found across right and left hemispheres in each individual subject, across the five individual datasets (Figs. 3 and 4, where pathways are represented as white wireframe volumes), and in agreement with previous results obtained from human *post-mortem* and NHP studies. Throughout the **Results** section, the connectivity patterns are described in a ventral-to-dorsal orientation.

#### Substantia nigra (SN)

SN is composed of two distinct parts, with *pars compacta* (SNc) located medially, and *pars reticulata* (SNr), laterally, within the nucleus. SN plays a central role in PD [44] and information regarding its connectivity patterns in individual patients is important for understanding better the mechanisms underlying this neurodegenerative disorder. Currently, such data is not available in human subjects. Figures 3*B-1* and 3*B-2* demonstrate reconstructions of the human nigrostriatal and nigropallidal pathways obtained *in-vivo* from two individual subjects. The **nigrostriatal** pathway (Fig. 3*B-1*) is particularly difficult to reconstruct—possibly because it is a longer-distance connection, and therefore affected by more fiber crossings. Note the consistency of the shape of the pathway across subjects in (Fig. 3*B-1*), and between left and right hemispheres of the same subjects. The **nigropallidal** pathway (Fig. 3*B-2*) provides dopaminergic projections to GPi, and evidence suggests that this pathway might be up-regulated in PD, with ongoing loss of nigrostriatal dopaminergic neurons [44]. We found that projections to GPi are mostly ventral (Fig. 3*B-2*), whereas nigrostriatal fibers project primarily to Pu medially, dorsally and ventrally (Fig. 3*B-1*). **Nigrothalamic** connections to putative thalamic nuclei, such as the medio-dorsal nucleus (MD), ventro-caudal nucleus (Vc), latero-polar nucleus (Lpo) and ventro-oral anterior nucleus (Voa) [45], [46], were also identified (Fig. 4*A*). These findings are consistent with results from NHP and rodent studies [47], [48] but have never been documented in humans.

#### Subthalamic nucleus

STN is a nodal component of the “indirect” pathway described above. The **subthalamopallidal** pathway has been proposed to regulate synchronized oscillatory burst activity in the basal ganglia [49]. We were able to reconstruct the projections between STN and GPe (Fig. 3*B-3*). Rostral GPe was found to connect with the medio-dorsal aspect of STN, while the ventro-caudal portion of GPe connected with the latero-caudal aspect of STN. These findings are supported by animal studies [50], where the massive projections from GPe to STN have been well-described [51]. Our results are also supported by a recent MRI population study performed at 1.5T [26], where data collected and averaged from eight human subjects identified subcortical connections of STN.

#### Globus pallidus

GP is composed of two distinct segments—an internal (GPi) and external (GPe) segment, separated by the *lamina pallidi medialis* [33] (Fig. 1). The ventral GP lies ventral to GPe, from which it is separated by the anterior commissure. Figure 3*B-4* depicts the **pallidothalamic** pathway, which is implicated in motor control and working memory [52]. Projections to thalamic nuclei—putatively identified as Lpo, the ventral-oral nucleus (Vo), centro-median parafascicular complex (CM/Pf), and MD—can be seen in Fig. 3*B-4*. We also successfully reconstructed the **striatopallidal** pathways—implicated in reward, aversion learning, and drug addiction [53]— with CN connecting to the dorso-medial third of GPe, and Pu to the ventro-lateral two-thirds of GPe (Figs. 4*B* and 4*C*, respectively). Similar connectivity patterns have been observed in animal studies [17], [54]. Pallidothalamic projections include the *ansa lenticularis* (AL) and the *lenticular fasciculus* (LF), and are demonstrated in (Fig. 3*B-4*). AL originates in the lateral portion of GPi, projecting in a ventral, medial and rostral direction, wrapping around the internal capsule toward putative CM/Pf, and entering the thalamic fasciculus (TF) toward putative Lpo, Voa, and the ventro-oral posterior nucleus (Vop) [19]. LF, although more difficult to visualize (Fig. 3*B-4*), originates in the medial portion of GPi, travels through the internal capsule and joins AL in TF [50]. The striatopallidal tract (shown in Figs. 4*B* and 4*C*) is the largest afferent projection to GPe. We find that ventral GP receives projections from a large striatal region and sends efferent tracts to STN (Fig. 3*B-3*), and thalamic nuclei (see Thalamus section below).

#### Striatum

As mentioned previously, the striatum receives most of the basal ganglia afferent projections from the cortex. We identified a medio-laterally decreasing gradient of projections to SN (Fig. 3*B-1*), with fewer projections identified in the dorso-lateral Pu than in the ventro-medial Pu, as reported in NHP [2]—although there is no clear consensus in the literature as to the location of striatal territories projecting to SN or GP. The striatum is known to send most of its efferent projections to both segments of GP, to SN, and to STN. It receives projections from CM/Pf, MD, Lpo and Vo. We identified **thalamostriatal** connections, projecting to the rostro-ventral and dorso-lateral CN (Fig. 4*D*), the loss of which has being shown to play a potential role in the striatal synaptic remodeling of PD [55].

#### Thalamus

Tha represents a complex cytoarchitectonic structure, with component nuclei projecting to a variety of different regions. The *cortical* connectivity of thalamic nuclei has been studied using tractography [20], [56], but thalamic *subcortical* projections have only recently been investigated in a study based on results from averaged human data [27]. In addition to the pallidothalamic, nigrothalamic and thalamostriatal pathways illustrated above, we found strong connections between the putative MD nucleus, especially its ventral portion, and ventral GP (Fig. 4*E*).

In summary, we successfully reconstructed and visualized, in individual subjects, the following seven critical pathways of the basal ganglia and thalamic connectome: nigrostriatal, nigropallidal, nigrothalamic, subthalamopallidal, pallidothalamic, striatopallidal, and thalamostriatal pathways.

### Anatomical parcellation of the basal ganglia and thalamic nuclei

A finer level of analysis was performed using a voxel-based approach, with the aims of (i) identifying anatomical subdivisions within each structure, and (ii) quantifying the probability of these connections (see Materials and Methods). This approach allowed for an investigation into the probabilities of pathways originating from a specified region-of-interest.

#### Substantia nigra

We identified subdivisions within SN that correlate with the known territories of SNr and SNc, and determined their connectivity. Figure 5*A* summarizes these findings: for all subjects, a separation of SN into a lateral and medial part is noted, putatively corresponding to SNr and SNc, respectively. The division between SNr and SNc is difficult to observe using current imaging techniques, but is nonetheless supported [57] by (i) histology, as SNr has a high iron content while SNc is melanized, and (ii) the segregated projections of each subdivision. We found that the most antero-lateral portion of SN was strongly connected to striatum, GPi, and GPe, while the postero-lateral portion was strongly connected to Tha. The medial portion of SN was found to be strongly connected to GPe and GPi, and to a lesser degree, to Tha (Fig. 5*A*). It was recently demonstrated that whole brain connectivity profiles from each voxel of SN allowed for the differentiation of SN into two parts, possibly corresponding to SNc and SNr [25]. Consistent with this study, we hypothesize that the medial SN, found to be connected to GP but not to the striatum, comprises part of SNc.

#### Subthalamic nucleus

We found that the dorso-lateral subdivision of STN is primarily connected to GP and Pu, while the ventro-medial part projects to SN and CN. Figure 5*B* shows the subdivisions of STN, based on their connections to GP (dorso-lateral motor territory) and SN (ventro-medial associative territory). Our findings agree with results from NHP [10]. Finally, the limbic territory of STN, situated in the antero-medial portion of the nucleus, projects to ventral and medial GP, and to CN [50], [57]. For clarity, striatal projections are not represented in this figure.

#### Globus pallidus

We were able to separate the medial, lateral and rostro-ventral parts of GPe as shown in Fig. 5*C*. The medial portion of GPe was found to connect to GPi, with its most dorsal and ventral portions connecting to SN. The lateral portion of GPe was noted to clearly connect with the medial portion of Pu. Finally, the most rostro-ventral portion of GPe was found to connect to Tha and STN, and the rostro-dorsal portion to CN. We also found clear subdivisions within GPi (see Fig. 5*D*), with its latero-caudal portion connected to Pu, its mid-portion connected to SN, and the rostral portion divided into a dorsal part connected to CN, and a ventral part connected to STN and Tha. The subdivisions of GPe and GPi shown here are consistent with recent results obtained by averaging data from thirty human subjects [27].

#### Striatum

Projections to the striatum are organized topographically, with a central associative, dorso-lateral sensorimotor, and ventromedial limbic territory receiving inputs from the cortex, Tha and SN [2]. We found that a large portion of central Pu and caudal CN connect to GPe (Figs. 5*E* and 5*F*, respectively). Finally, the striatal sensorimotor territory was found to connect mainly to SN and GPi (Figs. 5*E* and 5*F*). Such connectivity-based identification of striatal territories is supported by previously published results derived from NHP studies [2].

#### Thalamus

The representative topological subdivisions of the human thalamus are shown in Fig. 5*G* from two individual subjects. In general terms, the connectivity pattern was found, from caudal to rostral, to be: striatum, SN, STN, GPi and, more medially, GPe. Nuclei putatively identified as pulvinar, Lpo, and Voa were found to connect mainly to the striatum, while putative Vc connected to SN, and putative ventro-intermediate nucleus (Vim) to STN. Subdivisions identified as CM and Lpo demonstrated connections primarily with GPi (in green, see color code in Fig. 5*G*). Finally, massive thalamostriatal connections to CN were identified arising from putative Lpo, Voa, Vop, the dorso-intermediate nucleus (Dim) and MD nuclei (Fig. 4*D*), as reported in [27]. MD was also found to connect to ventral GP (Fig. 4*E*). Highly similar connectivity patterns were seen in all subjects. The findings described above are in excellent agreement with results of NHP studies, where it has been shown that GPi and SNr represent the primary basal ganglia inputs to thalamus (Lpo, Vo, Vim), and that ventral GP projects to MD [2]. Evidence for a direct projection to the ventral thalamus from STN has also recently been described in NHP [51].

### Probability of connections

The territories described above were identified by categorizing voxels of each structure as connected to another target structure if the voxels had a high proportion of probabilistic streamlines reaching the target in question. No grouping or segmentation techniques were used. Figure 6 summarizes the statistics, based on all datasets, of the average proportion (over each sub-territory) of streamlines connecting each pair of component structures of the basal ganglia and thalamus, and for each hemisphere (see details in Materials and Methods). As specific examples, left SN demonstrated strong connections with Tha (0.7, nigrothalamic pathway) and STN (0.5), and moderate connections with GP and striatum (0.3 to 0.4, nigropallidal and nigrostriatal pathways). There was a high degree of consistency between right and left hemispheres and across subjects, as is demonstrated in Fig. 6.

STN was found to connect preferentially and equally to SN and Tha (0.5), a finding which might reflect the spatial proximity of these structures. Connections of STN to GP (subthalamopallidal pathway) were slightly higher for GPi than for GPe, since projections to GPe have to traverse GPi. STN connections to striatum were weak.

GPi was found to connect strongly to Tha (0.6, pallidothalamic pathway), GPe (0.6), SN (0.5) and Pu (0.4). GPe demonstrated a similar connectivity profile to GPi and strong connections to Tha through ventral GP and Pu (striatopallidal “indirect” pathway).

The thalamus demonstrated consistently high probabilities of connection with all other structures. Additional results pertaining to the volumes of each sub-territory are available in (Fig. S1). A matrix representation of the probability of connection is shown in (Fig. S2) and emphasizes, for each hemisphere and each connection, the symmetry of these probabilities between the hemispheres.

### Functional connectivity via R-fMRI

The anatomical/structural connections described above were supported by evaluating the functional connectivity between these region-of-interests using resting-state fMRI. R-fMRI activation maps of each structure of interest were estimated using a seed-based approach [58], as described in Materials and Methods. Figure 7 demonstrates one such example of spatial agreement between anatomical/structural connectivity and functional connectivity mapping techniques. R-fMRI activated areas are displayed as colored isolines of equal statistical significance (*p*<0.01), and merged with the segmentations of regions-of-interest and white matter pathways. Figure 7*A* shows the nigrostriatal pathway (white wireframe volume) connecting SN (yellow) and Pu (red), overlaid with the R-fMRI activations map that correspond to regions with R-fMRI time courses strongly correlated with the mean time course of SN. A cluster of activated voxels was identified within Pu (red), at the location where part of the nigrostriatal tract ends, which is in good agreement with the spatial localization of the anatomical findings. Similarly, Fig. 7*B* demonstrates overlap between the endpoint of the nigral projections in thalamus, and the R-fMRI activations of SN within Tha.

### Asymmetries

Using a parametric t-test and a non-parametric Kolmogorov-Smirnov test (p<0.05), inter-hemispheric (left vs. right) asymmetries were found for the probability of connection between the following structures: GPe-Pu (Kolmogorov-Smirnov test) and SN-GPi (t-test and Kolmogorov-Smirnov test), as indicated in Fig. 6.

## Discussion

### Mapping the human basal ganglia and thalamic connectome

This work represents a comprehensive, *in vivo*, investigation of the human basal ganglia and thalamic connectome. Contrary to most existing studies, which are based on animal data, histology, or group-averaged MR imaging of human subjects (Table 1), the data presented here was acquired in individual human subjects.

The imaging and analysis protocol used in this study has yielded consistent results across subjects, between left and right hemispheres in each individual subject, and between different imaging modalities. The high spatial resolution afforded by 7T MRI enables the clear delineation of structures such as GPe, GPi, STN, and SN [33]. It also enables the detailed reconstruction of the white matter tracts that comprise the basal ganglia and thalamic circuitry, and a quantification of the strength of these connections. We successfully parcellated the basal ganglia and thalamus into distinct anatomical territories on the basis of their projections, and quantified the probability of these connections and the spatial extent of these subdivisions. These findings are concordant with results obtained primarily in animal studies—such as the existence of (i) strong pallidothalamic projections, and (ii) the nigrostriatal pathway. Our findings also correlated with, and were supported by, R-fMRI data, which demonstrated overlap of anatomical and functional connectivity maps. Although functional connectivity does not necessarily imply direct anatomical connectivity, the detection of overlapping territories through both functional and anatomical techniques is a strong indication of the reliability of the proposed model.

**Figure 8:**
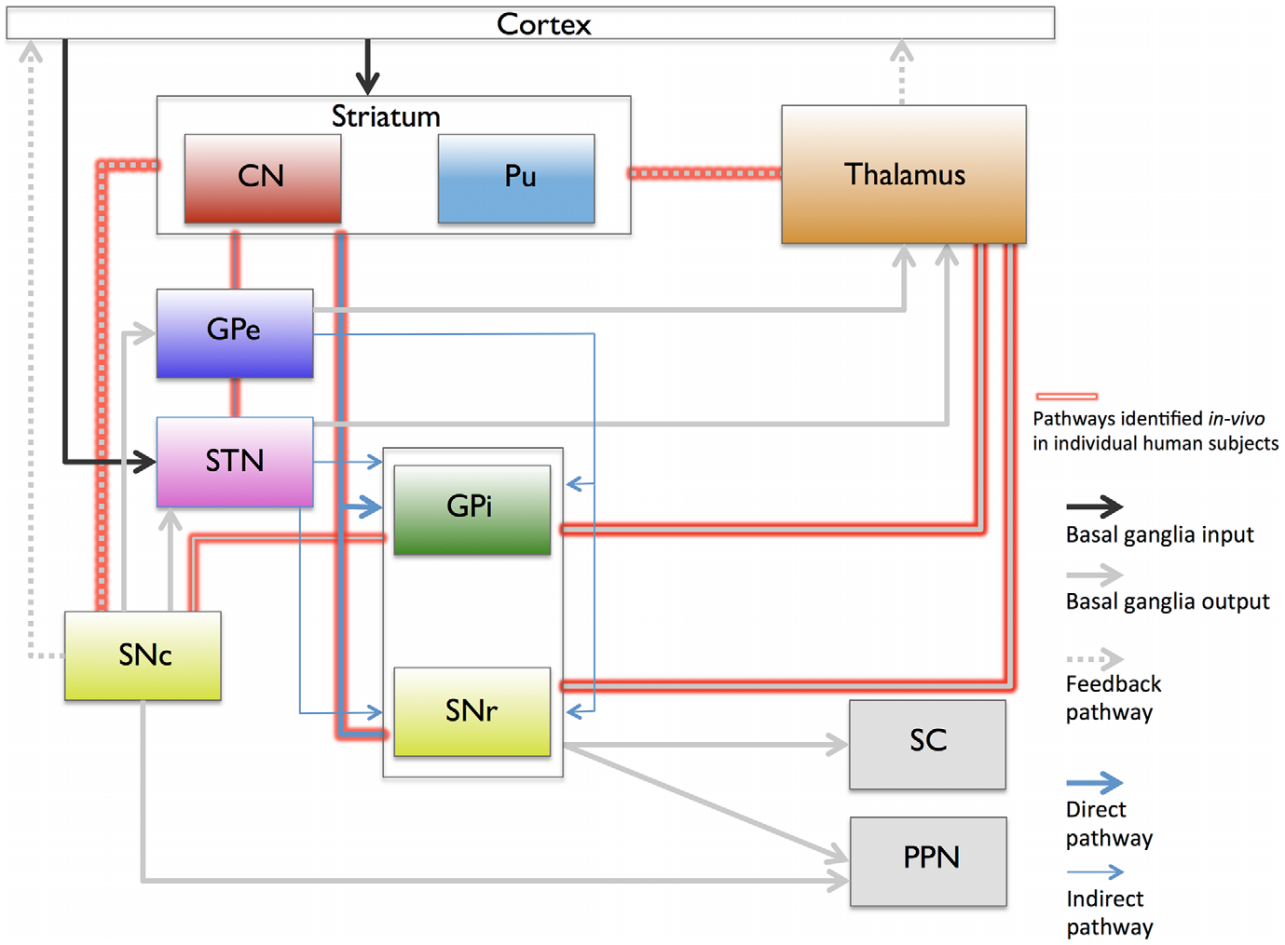
Basal ganglia and thalamus circuits, and newly identified pathways *in-vivo* in individual human subjects. The diagram, adapted from [7], summarizes known pathways of the basal ganglia and thalamus connectome, from histology and recent MRI population studies. The lines outlined in red indicate pathways uniquely identified *in-vivo* in individual human subjects in this study using 7T MRI. Although the directionality (afferent/efferent) of these pathways is known from histology, diffusion MRI tractography is unable to recover this information, hence the absence of polarity for the pathways identified in this study.

Based on the data presented here, we propose an updated description of the human basal ganglia and thalamic connectome, summarized in Fig. 8. The seven anatomical pathways we successfully visualized in individual living human subjects, are highlighted in red. These pathways are also summarized in Table 1, which provides the details of the previous studies used to identify the pathways. It is also worth noting here that diffusion MRI is unable to resolve the *polarity* of projections, making it impossible to differentiate between afferent and efferent connections. Directional information, indicated by arrows (Fig. 8), is only provided for the sake of completeness and is based on studies listed in Table 1. Nonetheless, this study lays the groundwork for future investigations into the mathematical properties of the networks constituted by white matter pathways and associated probabilities.

The unique datasets presented here allow for the exploration of the fine spatial relationships and connectivity patterns of brain structures, which are typically lost when images are averaged across multiple subjects, due to the limited sensitivity of the acquired data. The technique of group analysis—the pooling of data across subjects—arose out of a lack of sufficient SNR to resolve structures within individual subjects. With the advent of 7T imaging, sufficient SNR now exists to allow for the resolving of smaller structures without the need to average across multiple subjects. We have demonstrated here the strong reliability and reproducibility of our measurements both between hemispheres in a given subject, and between subjects. Nonetheless, as supported by our data (Error bars in Fig. 6 represent the standard deviation of the measurement across subjects) and as routinely observed in the operating room during DBS surgery, there are significant variations between individuals that justify the acquisition of subject-specific data over population data.

Another illustration of the richness of our datasets is the finding of inter-hemispheric asymmetries in two specific pathways such as the GPe-Pu and the SN-GPi connections. Although the significance of such asymmetries is currently unknown, this finding bears further investigation, as the SN-GPi pathway plays an important role in PD [44].

### Clinical relevance, future research and limitations

DBS surgery targeting various brain nuclei has become standard-of-care for the treatment of movement disorders, such as PD, essential tremor, and dystonia. Indications and targets for DBS are expanding, yet both the mechanism of DBS action, and the optimal target(s) for each indication, remain the subject of debate. Furthermore, imaging of these DBS targets and their three-dimensional surroundings has yet to be optimized [33]—an issue which, if resolved, could contribute to the optimization of DBS electrode positioning during surgery. The work presented here addresses some of these questions by providing detailed information about the connections of the human basal ganglia and thalamus. We have demonstrated, for example, reconstructions of the nigrostriatal pathway—a pathway which is crucial for the proper functioning of the basal ganglia circuitry, and when diminished, is known to result in hyperactivity of the basal ganglia output structures, leading to the signs and symptoms of PD [59].

Moreover, recent studies have reported clinical benefit from high-frequency stimulation of white matter areas adjacent to known DBS targets [60], [61]. In contrast to the traditional DBS targets, which are nuclear structures (e.g., STN, Vim, and GPi), these newer target areas are largely white matter regions containing pathways such as the pallidofugal and cerebellothalamic tracts. Stimulation of these tracts might represent a more potent means of modulating the neural networks that are dysfunctional in the movement and neuropsychiatric disorders. The fine mapping of the basal ganglia/thalamic connectome made possible by the techniques demonstrated here, may provide valuable pre-operative information about targeting specific structures and planning DBS surgery. This may, in turn, translate into improved clinical outcomes.

We must emphasize, nonetheless, certain limitations of this work. First, high-field imaging may be associated with greater field inhomogeneities and image distortions. These issues could hamper accurate identification of ROIs when attempting to co-register several imaging modalities, as proposed here. More specifically, eddy-current and susceptibility-induced distortions must be corrected in order to ensure proper co-registration of diffusion, functional, and structural MRI data (See section Materials and Methods). Although these effects were limited in our datasets, and corrections were carefully applied, future studies will need to consider this potential pitfall, as well as the accuracy of co-registration with clinical 1.5T MRI scans. Second, although images were acquired at high spatial resolution, certain pathways (such as the subthalamo-thalamic tract) could be estimated, and their strength quantified, but were not *visualized* because of the proximity of the structures involved. This issue could be addressed in future work by restricting acquisitions to these specific areas, with higher spatial resolution—at the cost of decreased SNR—and modified diffusion sampling schemes. Finally, this study provides new tools with which to investigate longitudinal alterations in basal ganglia and thalamic structure and connectivity in patients with movement and neuropsychiatric disorders, allowing for investigations into structural and functional changes at the level of overall target shape, volume, sub-territory organization, and in the strength of connections—and the clinical significance of any such findings.

### Conclusion

This work enables investigations into the anatomical and functional circuits of the human basal ganglia and thalamic connectome. Our results open new avenues into the investigation of (i) mechanism(s) underlying the movement disorders and neuropsychiatric disorders, and of (ii) therapeutic effects—and possible optimization—of interventions such as DBS.

## Materials and Methods

### Ethics statement

The research protocol used in this investigation was approved by the Institutional Review Board of the University of Minnesota. All subjects provided informed written consent prior to participating in the research. Subjects ranged in age from 23 to 57 years, and had no prior history of neurological disorders. Anatomical imaging for the purposes of this study revealed no structural abnormalities in any of the subjects.

### Data acquisition

Four healthy subjects were scanned at the Center for Magnetic Resonance Research of the University of Minnesota, using a 7T magnet. One subject was scanned twice on two different days, yielding a total of five datasets. The 7T MRI (Magnex Scientific, UK) is driven by a Siemens console (Erlangen, Germany), and uses a Siemens head-gradient insert capable of 80 mT/m in 135 msec. A 16-channel transmit/receive head coil was used, with the radiofrequency (RF) power split evenly between the channels.

The scanning protocol included T1-weighted and proton-density MRI, high-resolution T2-weighted MRI and susceptibility-weighted imaging (SWI) of the midbrain, whole-brain high-angular resolution diffusion imaging (HARDI) and resting-state functional MRI (R-fMRI). R-fMRI data from one subject was not usable because of excessive head motion and could not be included in our analysis.

- T1-weighted images were acquired with a standard Siemens 3D-MPRAGE sequence using the following parameters: FOV = 256×216×176 mm^3^, matrix size = 256×216×176 (1 mm^3^ resolution), repetition/inversion/echo time (TR/TI/TE) = 3000/1500/4.29 msec, flip angle = 4°, bandwidth (BW) = 140 Hz/pixel, with an acceleration factor of 2 (GRAPPA) along the phase-encoding direction. A proton-density weighted volume was acquired with parameters identical to the MPRAGE acquisition. Total acquisition time was approximately 6 min.
- T2-weighted images were acquired with a 2D turbo spin echo sequence using the following parameters: FOV = 205×205×36 mm^3^, matrix size = 512×512×18 (0.4×0.4×2.0 mm^3^ resolution), TR/TE = 5000/57 msec, flip angle = 120°, BW = 220 Hz/pixel, with an acceleration factor of 2 (GRAPPA) along the phase-encoding direction. The total acquisition time was approximately 7 min for one average. This protocol was repeated twice, to obtain both axial and coronal images of the midbrain.
- Susceptibility-weighed images (SWI) were acquired with a 3D flow-compensated gradient echo sequence using the following parameters: FOV = 180×180×60 mm^3^, matrix size = 448×448×60 (0.4×0.4×1.0 mm^3^ resolution), TR/TE = 28/20 msec, flip angle = 15°, BW = 120 Hz/pixel, with an acceleration factor of 2 (GRAPPA) along the phase-encoding direction. One average was used, for a total acquisition time of approximately 7 min. This protocol was also repeated twice, to obtain both axial and coronal images of the midbrain. Figure 1 shows examples of high-resolution T2-weighted and SWI images in two subjects.
- Diffusion MRI was acquired with a single refocused 2D single-shot spin echo EPI sequence [62] using the following parameters: FOV = 192×192×99 mm^3^, matrix size = 128×128×66 (1.5×1.5×1.5 mm^3^ resolution), TR/TE = 5000/50 msec, flip angle = 90°, BW = 2440 Hz/pixel, with an acceleration factor (GRAPPA) of 3. Diffusion-weighted images (*b*-value = 1500 s/mm^2^) were acquired with diffusion gradients applied along 128 uniformly distributed directions [63]. Fifteen additional non-diffusion-weighted images (*b* = 0 s/mm^2^) were acquired every 10 diffusion-weighted images, for a total acquisition time of 12 min.
- Resting-state blood oxygenation level dependent (BOLD) functional MRI was acquired with a 2D single-shot gradient echo EPI sequence using the following parameters: FOV = 192×192×99 mm^3^, matrix size = 128×128×66 (1.5×1.5×1.5 mm^3^ resolution), TR/TE = 2000/17 msec, flip angle = 90°, BW = 2440 Hz/pixel, with an acceleration factor of 3 (GRAPPA). 150 time frames were acquired for a total time of approximately 5 min.
- Field maps were acquired with a 2D single-shot gradient echo sequence, using the same FOV and resolution as diffusion and resting-state functional MRI. Two complex images were acquired with echo times = 5.1 and 6.12 msec, TR = 514 msec, flip angle = 30° and BW = 795 Hz/pixel. In order to ensure optimal alignment with the EPI data, field maps acquisitions were repeated immediately before the diffusion-weighed MRI and the R-fMRI series.

### Pre-processing of diffusion MRI

**Figure 9:**
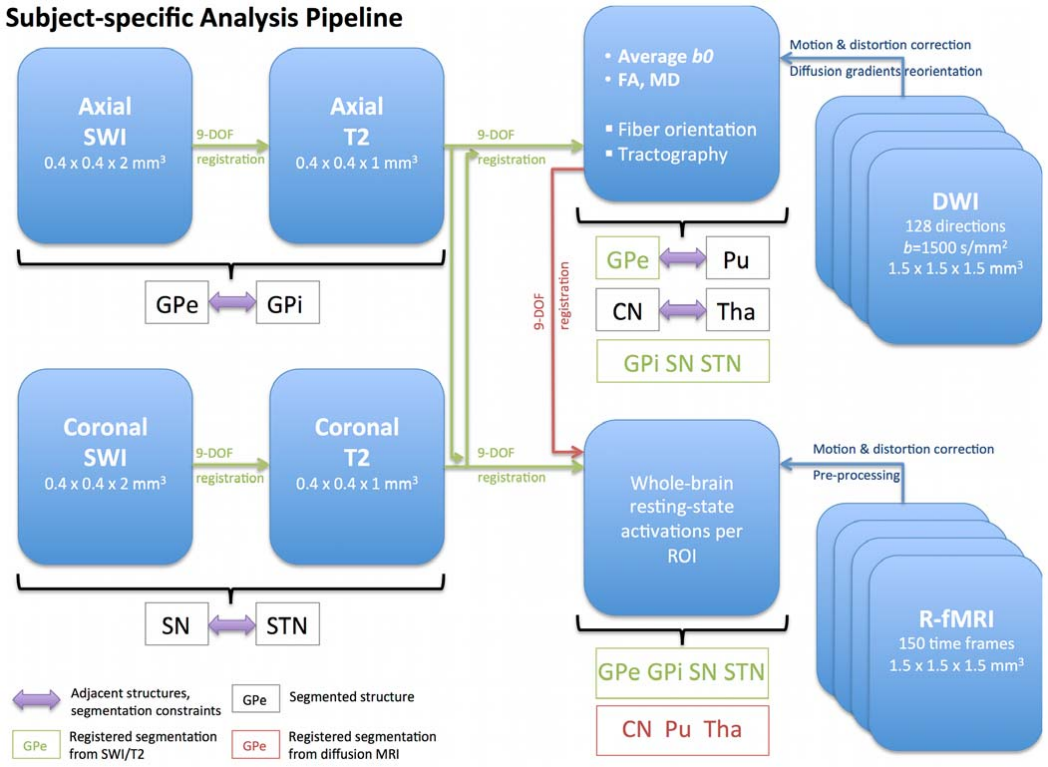
Subject-specific analysis pipeline. This diagram summarizes the pre-processing, registration, and segmentation steps that were required to identify the seven regions-of-interest used in this work: caudate nucleus (CN), putamen (Pu), external and internal globus pallidus (GPe and GPi), substantia nigra (SN), subthalamic nucleus (STN), and thalamus (Tha). Axial SWI and T2-weighted images were linearly registered using nine degrees-of-freedom (DOF), and used to delineate GPe and GPi. Coronal SWI and T2-weighted images were also linearly registered using nine DOF, and used to delineate SN and STN. The purple arrows indicate spatial relations between structures, exploited to segment them (i.e. GPe and GPi are adjacent and simultaneously segmented). Diffusion-weighted images were corrected for motion, eddy-current, and susceptibility-induced distortions, and were then used to estimate fractional anisotropy (FA) and averaged *b0* images. Subsequently, axial and coronal high-resolution T2-weighted images were aligned with the averaged *b0* in order to resample segmentations of GPe, GPi, SN, and STN into diffusion native space, and then used for tractography seeding. CN, Pu and Tha were segmented from the FA image. Finally, resting-state fMRI (R-fMRI) data were corrected for motion and susceptibility-induced distortions. An average R-fMRI image was created and used as a template to register axial and coronal T2-weighted images, and *b0*, in order to resample all regions-of-interest into R-fMRI native space and then to generate R-fMRI activation maps.

Analysis of diffusion-weighted images was performed in native space using FSL 4.1.6 (Analysis Group, FMRIB, Oxford, UK) [64], [65]. For each subject, diffusion-weighted images were first corrected for head motion and eddy current distortions using *flirt* [66] linear registration with 12 degrees of freedom (DOF). Individual transformation matrices for each diffusion-weighted image were used to reorient the corresponding diffusion gradient before any model fitting, such as diffusion tensor, or crossing fibers estimation. Subsequently, images were corrected for susceptibility-induced geometric distortions with *fugue*, using an unwrapped field map generated by *prelude* [67]. Corrected images were visually inspected for good alignment with T1-, T2- and susceptibility-weighted images. Color fractional anisotropy (FA) maps [68] were estimated. Non-diffusion-weighted images were averaged to obtain a high-SNR reference image for inter-modalities registration (see Fig. 9).

### Pre-processing of resting-state functional MRI

Analysis of R-fMRI data was performed in native space, using BrainVoyager QX 2.2 (Brain Innovation, The Netherlands) [69]. The first volume was discarded, yielding 149 time frames with an inter-slice time of 30 msec. Global signal fluctuations were corrected by adjusting the mean intensity of each slice according to the reference (first) volume. Slice-scan time correction was performed in order to account for the delay between the acquisition of the first and last slice of each volume. Head motion was estimated and corrected using a rigid transformation with 6 DOF. Estimated motion for all but one subject (which was discarded from the analysis) was less than 1 mm or 1 degree in all three directions. Temporal high-pass filtering using a general linear model (GLM), with a Fourier basis set consisting of 2 since/cosine pairs, was applied separately to each voxel to correct for linear and non-linear signal drift. Finally, spatial and temporal smoothing was performed to improve signal-to-noise ratio (SNR) and statistical power. Spatial smoothing with 3D Gaussian kernel of 4 mm full width at half maximum (FWHM) and temporal smoothing with Gaussian kernel of two data points was used. Subsequently, as for the diffusion-weighted images, geometric distortions were corrected using an unwrapped field map (see Fig. 9).

### Segmentations of the basal ganglia and thalamus

Fourteen regions-of-interest (ROI) were manually segmented in each of the five datasets (see example for Subject 1 in Fig. 3*A*). These consisted of seven ROIs per hemisphere, including CN, Pu, GPe, GPi, SN, STN and Tha. In order to minimize errors in segmentation, each ROI was delineated in all datasets (left hemisphere first, then right hemisphere) before moving to the next ROI. High-resolution axial and coronal T2-weighted images were aligned with the corresponding SWI using 9 DOF linear registration. Both modalities were simultaneously used to identify SN, STN, GPe and GPi. Axial images were used to delineate the segments of GP since images in this plane provided optimal visualization of the *lamina pallidi medialis* which separates GPi from GPe. Coronal images were used to segment SN and STN since images in this plane allowed for the best separation of STN and SN along the lateral-medial axis (see Fig. 1). FA maps were used to delineate CN, Tha and Pu, using resampled GPe segmentation to identify the boundary between Pu and GP.

All ROIs were resampled from their respective native space into diffusion or functional MRI spaces, for subsequent analysis, by aligning T2-weighted onto averaged non-diffusion-weighted images, T2-weighted onto averaged functional images, and averaged non-diffusion-weighted onto averaged functional images. Figure 9 summarizes the various steps involved in data pre-processing, registration and segmentation.

### Probabilistic tractography

Fiber orientations, and their volume fraction and dispersion, were estimated at each voxel using FSL *bedpostx* with a maximum number of three crossing fibers. Probabilistic tractography [70], which exploits fiber orientation information to approximate the three-dimensional configuration of fiber tracts by propagating streamlines in a probabilistic fashion, was used to estimate the connectivity distribution of white matter pathways between each pair of ROIs (21 per hemisphere). It was also used to parcellate the basal ganglia and thalamus into distinct subdivisions based on their connectivity profiles.

#### Pathway identification

Connectivity distributions count, for each voxel of the imaged volume, the number of probabilistic streamlines running between an entire reference ROI and an entire target ROI through this specific voxel. These were normalized by the total number of streamlines reaching each target ROI (*i.e.*, streamlines reaching the rest of the brain were discarded) and could thus be compared. The closer a given probability is to 1, the more probable it is that a specific voxel belongs to the estimated pathway. Voxels with a proportion of streamlines greater than 10% of the maximum value of a given connectivity distribution were retained as part of the pathway of interest.

#### ROI parcellations

Probabilistic tractography was also performed from each voxel of each ROI, in order to quantify the probability of its connection with any of the other structures of interest. Voxels with a proportion of streamlines to another structure measuring >50% of the maximum value of the connectivity distribution, were categorized as part of the subdivision connected to that structure. We measured the volume of these subdivisions, and computed statistics of the connectivity values within these subdivisions to quantify their probability of connection with other structures (Fig. 6). For example, 40% (±8% over the five datasets) of the streamlines drawn from each voxel of the sub-territories colored red on Fig. 5*E* reached Pu. Ten percent (±5%) of the streamlines drawn from each voxel of the sub-territories colored yellow reached SN. These proportions indicate the relative probability of connections of each structure. A value of 30% might seem low, but reflects SNR, fiber crossings, image resolution, etc., and has to be compared to other proportions.

### Statistical analysis of R-fMRI

Resting-state functional connectivity maps were obtained for each structure of interest as follows: BrainVoyager QX 2.2 was used to run a univariate General Linear Model (GLM) analysis of the R-fMRI data with a seed-based approach [58]. Voxel time courses were regressed against the mean time course of each ROI, thereby producing whole-brain functional connectivity of the basal ganglia and thalamus. The mean brain time-course, motion correction, and mean intensity adjustment parameters were added as confounding factors to the design matrix. False Discovery Rate (FDR) [71] was used to determine functionally correlated voxels at *q*<0.01. Clusters of at least 10 voxels were retained in our analysis.

## Supporting Information

Figure S1 **Volumes of sub-territories of the basal ganglia and thalamus identified from their white matter projections.** This chart provides mean and standard deviations of the volume (proportions of the whole region) occupied by each sub-territory in each region-of-interest, over the five datasets. Sub-territories are identified in the basal ganglia and thalamus by exploiting the fact that these divisions exhibit distinctively stronger connectivity (than other areas of the same region-of-interest) with other structures. Some large proportions can be explained by the spatial proximity of structures. For instance, the high percentage of territory within CN that is allotted to Tha might be more reflective of the proximity of these two structures than of the actual size of the sub-territory within CN that is occupied by thalamic white matter projections. It should be noted, however, that the subthalamic nucleus is extensively connected to all other structures, which is consistent with its nodal role in the indirect pathway. Color code: Caudate nucleus, CN: light blue; Putamen, Pu: red; External globus pallidus, GPe: dark blue; Internal globus pallidus, GPi: green; Substantia nigra, SN: yellow; subthalamic nucleus, STN: magenta; Thalamus, Tha: orange.
(TIF)

Figure S2 **Probability of connection between basal ganglia and thalamus.** This matrix provides another representation of the data contained in Fig. 6, and emphasizes the symmetry of the probability values, within each hemisphere, as well as the strong agreement between hemispheres. Note the strong connection of the thalamus with each structure of the basal ganglia. Color map: Proportion of probabilistic streamlines starting from a given structure and reaching a specific target region, by comparison with the total number of streamlines reaching the entire basal ganglia area or thalamus (c.f. Fig. 6).
(TIF)
