# Supplementary figures and images for: Comprehensive in vivo Mapping of the Human Basal Ganglia and Thalamic Connectome in Individuals Using 7T MRI
Source: PLoS One. 2012 Jan 3;7(1):e29153. doi: 10.1371/journal.pone.0029153 (PMC3250409; doi:10.1371/journal.pone.0029153)

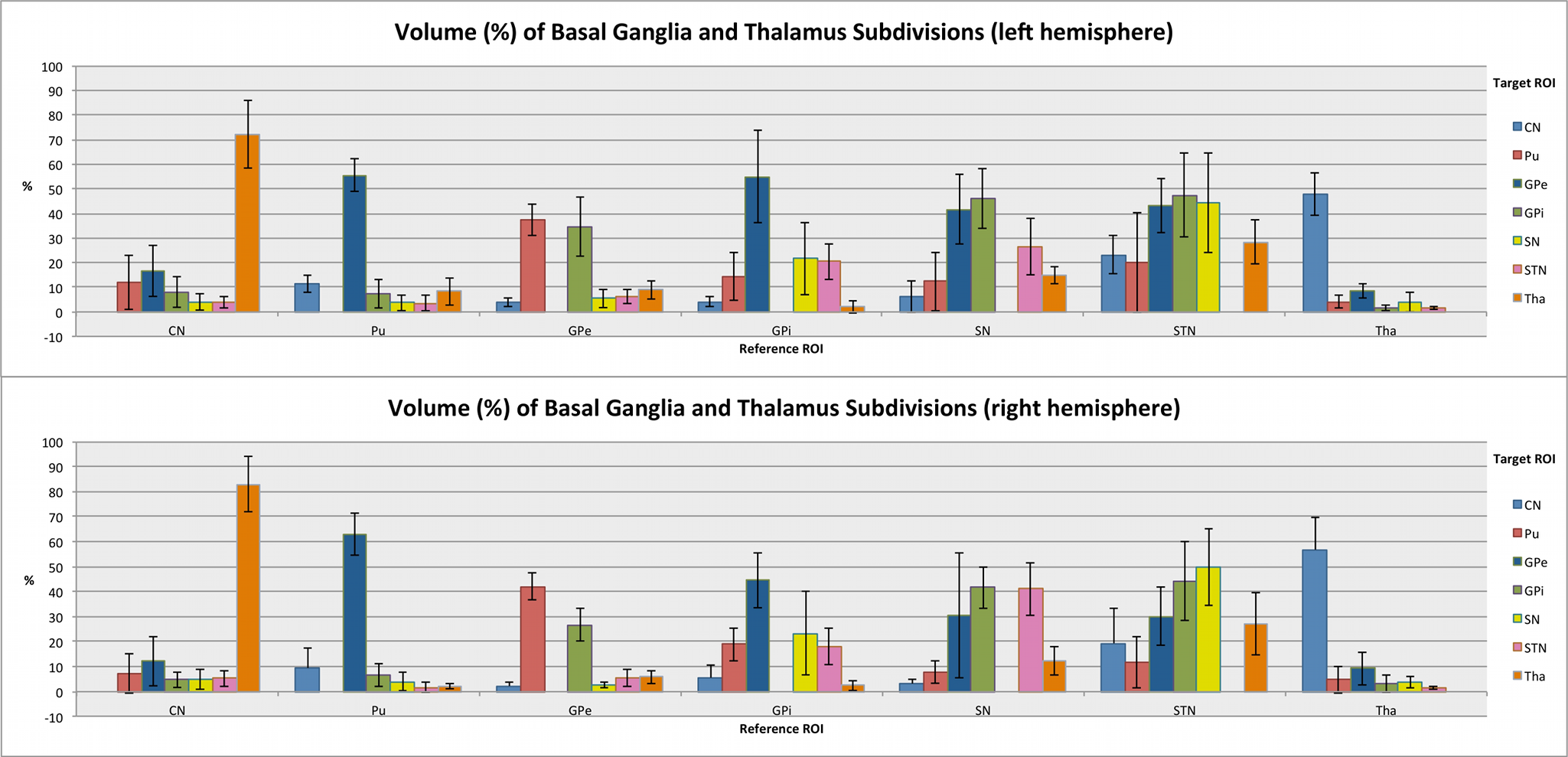

Supplement: Figure S1 — Volumes of sub-territories of the basal ganglia and thalamus identified from their white matter projections. This chart provides mean and standard deviations of the volume (proportions of the whole region) occupied by each sub-territory in each region-of-interest, over the five datasets. Sub-territories are identified in the basal ganglia and thalamus by exploiting the fact that these divisions exhibit distinctively stronger connectivity (than other areas of the same region-of-interest) with other structures. Some large proportions can be explained by the spatial proximity of structures. For instance, the high percentage of territory within CN that is allotted to Tha might be more reflective of the proximity of these two structures than of the actual size of the sub-territory within CN that is occupied by thalamic white matter projections. It should be noted, however, that the subthalamic nucleus is extensively connected to all other structures, which is consistent with its nodal role in the indirect pathway. Color code: Caudate nucleus, CN: light blue; Putamen, Pu: red; External globus pallidus, GPe: dark blue; Internal globus pallidus, GPi: green; Substantia nigra, SN: yellow; subthalamic nucleus, STN: magenta; Thalamus, Tha: orange. (TIF) [file pone.0029153.s001.tif]

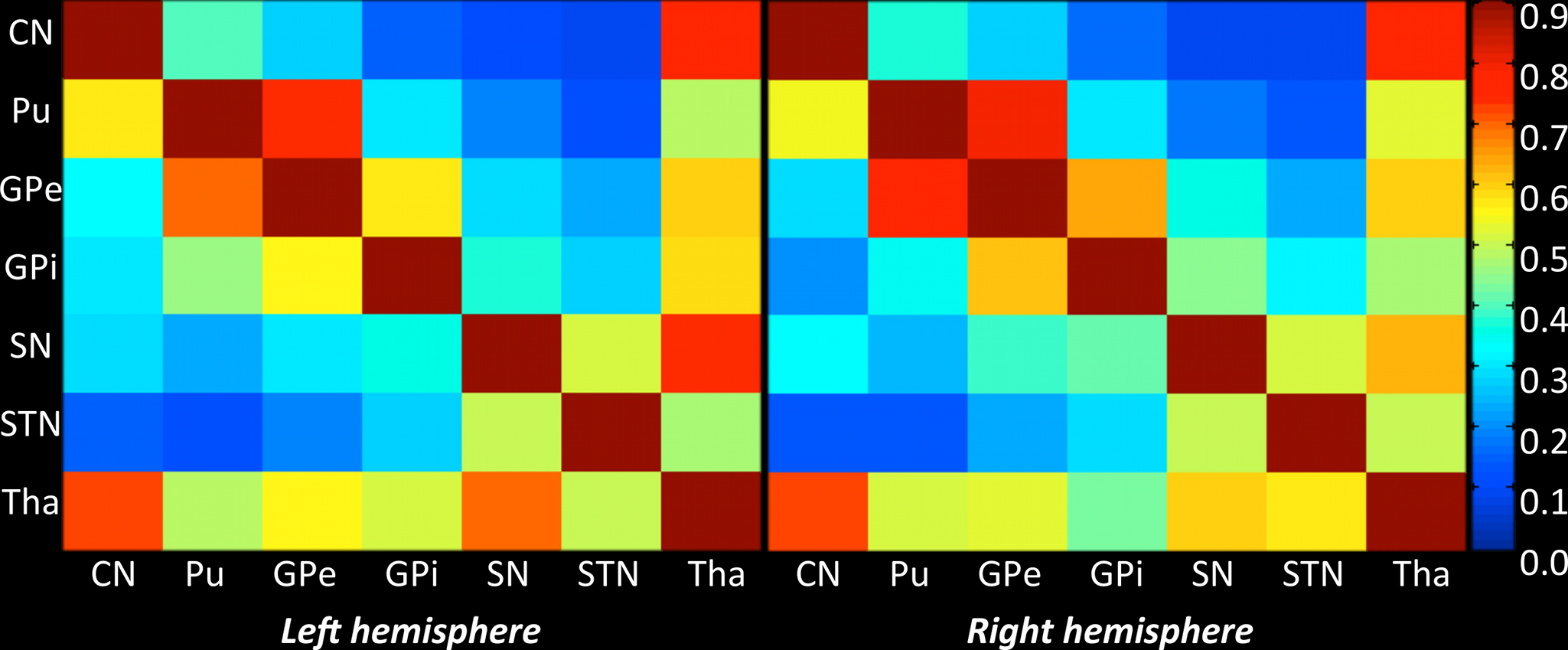

Supplement: Figure S2 — Probability of connection between basal ganglia and thalamus. This matrix provides another representation of the data contained in Fig. 6, and emphasizes the symmetry of the probability values, within each hemisphere, as well as the strong agreement between hemispheres. Note the strong connection of the thalamus with each structure of the basal ganglia. Color map: Proportion of probabilistic streamlines starting from a given structure and reaching a specific target region, by comparison with the total number of streamlines reaching the entire basal ganglia area or thalamus (c.f. Fig. 6). (TIF) [file pone.0029153.s002.tif]
